# Risk prediction of pulmonary tuberculosis using genetic and conventional risk factors in adult Korean population

**DOI:** 10.1371/journal.pone.0174642

**Published:** 2017-03-29

**Authors:** Eun Pyo Hong, Min Jin Go, Hyung-Lae Kim, Ji Wan Park

**Affiliations:** 1 Department of Medical Genetics, College of Medicine, Hallym University, Chuncheon-si, Ganwon-do, Republic of Korea; 2 Center for Genome Science, National Institute of Health, Cheongju-si, Chungcheongbuk-do, Republic of Korea; 3 Department of Biochemistry, School of Medicine, Ewha Womans University, Seoul, Republic of Korea; South Texas Veterans Health Care System, UNITED STATES

## Abstract

A complex interplay among host, pathogen, and environmental factors is believed to contribute to the risk of developing pulmonary tuberculosis (PTB). The lack of replication of published genome-wide association study (GWAS) findings limits the clinical utility of reported single nucleotide polymorphisms (SNPs). We conducted a GWAS using 467 PTB cases and 1,313 healthy controls obtained from two community-based cohorts in Korea. We evaluated the performance of PTB risk models based on different combinations of genetic and nongenetic factors and validated the results in an independent Korean population comprised of 179 PTB cases and 500 healthy controls. We demonstrated the polygenic nature of PTB and nongenetic factors such as age, sex, and body mass index (BMI) were strongly associated with PTB risk. None of the SNPs achieved genome-wide significance; instead, we were able to replicate the associations between PTB and ten SNPs near or in the genes, *CDCA7*, *GBE1*, *GADL1*, *SPATA16*, *C6orf118*, *KIAA1432*, *DMRT2*, *CTR9*, *CCDC67*, and *CDH13*, which may play roles in the immune and inflammatory pathways. Among the replicated SNPs, an intergenic SNP, rs9365798, located downstream of the *C6orf118* gene showed the most significant association under the dominant model (OR = 1.59, 95% CI 1.32–1.92, *P* = 2.1×10^−6^). The performance of a risk model combining the effects of ten replicated SNPs and six nongenetic factors (i.e., age, sex, BMI, cigarette smoking, systolic blood pressure, and hemoglobin) were validated in the replication set (AUC = 0.80, 95% CI 0.76–0.84). The strategy of combining genetic and nongenetic risk factors ultimately resulted in better risk prediction for PTB in the adult Korean population.

## Introduction

Pulmonary tuberculosis (PTB) is a prevalent infectious disease that is caused by *Mycobacterium tuberculosis* (*M*. *tuberculosis*). According to the Global Tuberculosis Report published by the World Health Organization in 2015, the estimated numbers of new tuberculosis cases and tuberculosis deaths worldwide reached almost 9.6 million and 1.1 million in 2014, respectively. The incidence and mortality rate of PTB reported in South Korea (86 patients and 3.8 deaths per 100,000 populations, respectively) are higher than those identified in other developed countries. (http://www.who.int/tb/publications/global_report/en/).

There is no effective vaccine for PTB, and the efficacy of the *M*. *bovis* bacilli Calmette-Guérin (BCG) vaccine at preventing PTB has been estimated at approximately 50%. Additionally, anti-tuberculosis drugs can cause side effects, and multi-drug resistant PTB is of increasing global concern. Although one-third of the world’s population, approximately 2 billion people, are estimated to be infected with *M*. *tuberculosis* (latent TB infection), data suggest that only 5–10% of infected individuals develop clinical PTB later in life, indicating that genetic heterogeneity may confer differential susceptibility to infection [1]. Previous population-based studies have reported that individuals, particularly men, with nongenetic risk factors, such as human immunodeficiency virus (HIV) infection, diabetes mellitus (DM), cigarette smoking, alcohol consumption, and low body mass index (BMI, less than 20 kg/m^2^), may be more susceptible to PTB [2, 3].

A number of genes associated with host susceptibility to PTB, including *CCL2*, *IFNG*, *NOS2A*, *SLC11A1*, *SP110*, *TLR1*, and *TNF-α*, have been suggested in previous linkage and/or candidate gene studies. Specifically, the human leukocyte antigen (*HLA*) genes have been reported to be associated with PTB in multi-ethnic populations [4]. Early genome-wide association studies (GWASs) have reported two single nucleotide polymorphisms (SNPs), one located in the gene desert region of 18q11.2 (rs4331426) and the other in the Wilms tumor 1 (*WT1*) gene (rs2057178, 11p13), to be associated with PTB in African populations [5, 6]. While none of these SNPs achieved a genome-wide level of significance, eight new loci have demonstrated nominal significance in Indonesian and Russian populations [7], and a meta-analysis of studies conducted in Thai and Japanese populations suggested that a SNP near the *HSPEP1-MAFB* gene, rs6071980, was associated with PTB [8]. Subsequently, the association between PTB and the rs2057178 SNP in 11p13 has been replicated in African and Russian populations [6–9]. Curtis et al. (2015) reported associations between intronic SNPs in the *ASAP1* gene (8q24) and PTB in their Russian GWAS and provided further evidence of the role of *ASAP1* in PTB pathogenesis [10]. A recent large scale Icelandic GWAS (2016) reported that only three SNPs located within the *HLA* class II region were significantly associated with PTB infection and could not replicate associations between PTB and genes reported by previous GWASs [11]. This lack of replication could be attributable to genetic heterogeneity across study populations, underpowered GWASs, and/or small effect sizes of PTB susceptibility variants.

Recent studies have focused on the use of polygenic models to resolve the problem of missing heritability and concerns relating to the clinical utility of GWAS results. Using these models, even if no single marker is found to be significantly associated with a complex trait, a polygenic score combining the effects of multiple genetic markers can be used to demonstrate that a disease has a strong genetic basis [12, 13]. Complex diseases such as PTB occur as a result of the interplay between environmental exposures and many genetic variants, each potentially exhibiting only a small effect. Therefore, multiple genetic and environmental risk factors must be considered simultaneously to predict the risk of developing PTB [14, 15]. To the best of our knowledge, no GWAS, let alone GWAS-based predictive modeling, has been performed for PTB risk in Koreans.

In this study, we initially performed a GWAS using data from two community-based cohorts in Korea and then developed various risk models, each of which consisted of a different combination of genetic and nongenetic risk factors, to predict the risk of PTB. Finally, we validated the GWAS results and evaluated the predictive ability of our risk models using an independent Korean population.

## Materials and methods

### Study populations, data collection, and genotyping

A total of 467 cases with PTB and 1,313 healthy controls were selected from 10,004 participants aged 40 to 70 years included in two community-based cohorts, Ansung and Ansan, developed as part of the Korean Association Resource Project (KARE) [16]. The cases included individuals with a history of PTB, treatment, or medication. Patients with PTB were diagnosed by either a positive sputum smear or culture [17]. The controls were healthy individuals who did not report any history of disease, treatment, and medication. Cases and controls were not matched on any selected characteristic. Data for demographic, environmental, and clinical factors were collected through self-reported questionnaires and laboratory tests. A total of 352,228 SNPs out of all the SNPs genotyped using the Affymetrix Genome-Wide Human SNP array 5.0 (Affymetrix Inc., Santa Clara, CA, USA) passed the preliminary quality control test for inclusion in subsequent analyses (i.e., genotype call rates > 95% for both SNPs and individuals, minor allele frequency (MAF) > 1%, and Hardy-Weinberg *P*-value > 1×10^−6^). To reduce the probability of a potential bias due to missing values, we also included ~4.4 million SNPs that were imputed based on the East Asian reference panel of the 1000 Genomes Project (imputation accuracy, *r*^*2*^ > 0.8) using Minimac software [18].

The replication study included 179 cases and 500 healthy controls ascertained from 3,703 participants aged 40 to 70 years who participated in the Health Examinee (HEXA) cohort [19]. Among 1,473 individuals who met the eligibility criteria for healthy controls, we further excluded individuals with SBP > 120 mmHg, DBP > 80 mmHg, or high fasting glucose ≤ 100 mg/dl from subsequent analysis. A total of 646,062 SNPs remained after quality control exclusions from the SNPs genotyped using the Affymetrix SNP array 6.0 (Affymetrix Inc., Santa Clara, CA, USA). This research was approved by the Institutional Review Board of Hallym University (HIRB-2016-066), and all participants provided written informed consent. Further details regarding the study design and data collection methods used in the KARE and HEXA studies have been described elsewhere [16, 19].

### Statistical analysis

We evaluated the associations between PTB and 17 conventional risk factors, including household income, cigarette smoking, alcohol consumption, and 12 clinical variables using generalized linear regression models constructed using STATA v.11.2 (Stata Corp., TX, USA). Continuous variables, such as BMI, SBP, DBP, Hb, and BUN, were converted into categorical variables and the new categorical variables were analyzed as well.

We conducted a GWAS for PTB under three genetic models (i.e., additive, dominant, and recessive) after adjusting for age, sex, and BMI using PLINK v.1.07 (http://pngu.mgh.harvard.edu/~purcell/plink/) [20]. The 374 SNPs with *P* values less than 0.001 identified in the KARE GWAS were validated among subjects of the HEXA Study. In addition, we compared our results with findings cataloged in existing genetic association studies databases, such as the HuGE Navigator Phenopedia (https://phgkb.cdc.gov/HuGENavigator) and NHGRI-EBI GWAS Catalog (https://www.ebi.ac.uk/gwas). We evaluated the statistical power and the sample size to achieve sufficient statistical power (80%) for each SNP using the Genetic Power Calculator, under the assumptions; PTB prevalence of 15% in Koreans, the genetic model showing the lowest *P*-value for each SNP, complete LD, 1:2.81 case-control ratio used in the current study, and type 1 error rate at genome-wide significance level of 5×10^−8^ (http://pngu.mgh.harvard.edu/~purcell/gpc/).

### *In silico* functional analysis

After removing redundant SNPs (*r*^*2*^ > 0.8), we conducted *in silico* functional analyses of the ten replicated SNPs to evaluate if they are in high linkage disequilibrium with functional SNPs such as nonsynonymous SNPs, nonsense SNPs, and SNPs creating transcription factor binding sites, splicing regulator, or predicted miRNA binding sites using the SNP Function Prediction program of the SNPinfo Web Server (http://snpinfo.niehs.nih.gov/snpinfo/snpfunc.htm).

### Risk prediction for pulmonary tuberculosis

We generated a genetic risk score model comprising ten SNPs replicated in the HEXA study population. We developed weighted genetic, nongenetic, and combined risk score models to predict the risk of developing PTB (i.e., wGRS, wnGRS, and wGRS+wnGRS, respectively). For each individual, a wGRS was calculated by summing the values obtained from multiplying model-specific genotype scores (additive, 0, 1, 2; dominant, 0, 1, 1; recessive, 0, 0, 1) by the natural log transformed odds ratio (ln OR) for each SNP in the model. Likewise, four different wnGRS models composed of different combinations of the nine covariates (age, sex, BMI, systolic and diastolic blood pressures (SBP, DBP), hemoglobin (Hb), and blood urea nitrogen (BUN), cigarette smoking, and alcohol consumption) that demonstrated significant associations with PTB in the univariate models were calculated by categorizing (i.e., 0, 1 or 0, 1, 2) and summing the ln ORs across variables. The predictive abilities of the wGRS and wnGRS models were evaluated by comparing the area under the receiver operating characteristic curves (AUC) using the *roctab* and *roccomp* commands in STATA. After stratification into quartiles based on the risk scores of each model, we also examined the associations between each risk score quartile and PTB risk. Finally, we compared the performance of the PTB risk models in HEXA dataset.

## Results

### Association of nongenetic factors

Ten of the 17 conventional risk factors were identified as significantly associated with increased risk of PTB in the KARE study dataset. PTB risk was higher in men than in women (OR = 2.03, 95% CI 1.64–2.52, *P* = 1.1×10^−10^) and in people aged 50 years and older than their younger counterparts (OR = 1.81, 95% CI 1.46–2.24, *P* = 5.3×10^−8^). Environmental factors, such as low household income, cigarette smoking, and alcohol consumption, were also identified as potent risk factors. While high BMI was observed to be a strong protective factor against PTB, elevated SBP, DBP, Hb, and BUN levels were associated with increased risk of PTB. The results obtained using the HEXA dataset were similar to those obtained using KARE; however, the associations between PTB and alcohol consumption and BUN were marginally significant, and household income was not significantly associated with PTB in the HEXA dataset (Table 1). The application of the strict selection criteria excluded a large proportion of controls with hypertension and high fasting glucose levels and 500 healthy normotensive nondiabetic controls were analyzed in the HEXA Study. As a result, the replication study yielded stronger effects for the covariates than the effects reported in the initial study. For instance, the OR of TB for current smokers was much higher in the HEXA data than the KARE data (OR = 2.72, 95% CI 1.67–4.46 vs. 1.52, 95% CI 1.38–2.07). Categorization of continuous covariates resulted in loss of power; especially the statistical significance disappeared after dichotomization of BUN (Table 1). Among these risk factors, gender, age, and BMI remained in the optimal model after stepwise selection was performed in the KARE Study dataset. Of the ten variables that were available and significant in the univariate models, four variables: gender, BMI, SBP, and DBP, remained in the optimal model after stepwise selection (Table A in S1 File).

**Table 1:** Univariate logistic regression analysis for associations between baseline characteristics and pulmonary tuberculosis in the KARE and HEXA Studies.

	KARE				HEXA				
Characteristics^a^	Case	Control	OR (95% CI)^b^	*P*^b^	Case		Control	OR (95% CI)^b^	*P*^b^
	(N = 467)	(N = 1,313)			(N = 179)		(N = 500)		
Male, N (%)	286 (61.2)	574 (43.7)	2.03 (1.64–2.52)	1.1×10^−10^^c^	104 (58.1)		124 (24.8)	4.20 (2.93–6.02)	5.0×10^−15^^c^
Age, N (%)									
< 50	216 (46.2)	799 (60.8)	1.00		67 (37.4)		302 (60.4)	1.00	
50 ≤	251 (53.8)	514 (39.2)	1.81 (1.46–2.24)	5.3×10^−8^^c^	112 (62.6)		198 (39.6)	2.55 (1.79–3.62)	1.8×10^−7^
Household income (million won), N (%)									
< 100	138 (29.9)	281 (21.7)	1.00		21 (11.7)		39 (7.8)	1.00	
100 ≤	324 (70.1)	1,016 (78.3)	0.65 (0.51–0.82)	4.0×10^−4^	133 (74.3)		371 (74.2)	0.67 (0.38–1.17)	0.159
Cigarette smoking, N (%)									
Nonsmoker	240 (51.8)	835 (63.8)	1.00		110 (61.5)	406 (81.2)		1.00	
Ex-smoker	94 (20.3)	177 (13.5)	1.85 (1.38–2.47)	3.0×10^−5^	34 (19.0)	46 (9.2)		2.72 (1.67–4.46)	6.1×10^−5^
Current smoker	129 (27.9)	296 (22.6)	1.52 (1.18–1.95)	0.001	34 (19.0)	46 (9.2)		2.72 (1.67–4.46)	6.1×10^−5^
Alcohol consumption, N (%)									
Nondrinker	185 (39.7)	647 (49.4)	1.00		78 (43.6)	265 (53.0)		1.00	
Ex-drinker	39 (8.4)	69 (5.3)	1.98 (1.29–3.02)	0.002	9 (5.0)	16 (3.2)		1.91 (0.81–4.49)	0.138
Current drinker	242 (51.9)	594 (45.3)	1.42 (1.14–1.79)	0.002	90 (50.3)	219 (43.8)		1.40 (0.98–1.99)	0.063
BMI, kg/m^2^	23.45±0.13	24.70±0.08	0.86 (0.82–0.89)	1.3×10^−14^^c^^,^^d^	22.94±0.20	22.96±0.12		0.99 (0.93–1.06)	0.905^c^^,^^d^
< 25	338 (72.4)	734 (55.9)	1.00		137 (76.5)	393 (78.6)		1.00	
25–30	117 (25.0)	515 (39.2)	0.49 (0.39–0.63)	6.4×10^−9^	41 (22.9)	101 (20.2)		1.16 (0.77–1.76)	0.469
30 ≤	12 (2.6)	64 (4.9)	0.41 (0.22–0.76)	0.005	1 (0.6)	6 (1.2)		0.48 (0.06–4.01)	0.496
SBP, mmHg	119.06±0.87	116.15±0.44	1.01 (1.01–1.02)	0.001^d^	122.80±1.09	107.47±0.37		1.17 (1.14–1.21)	4.1×10^−27^^c^^,^^d^
< 120	296 (63.4)	952 (72.5)	1.00		70 (39.1)	500 (100)			
120–140	117 (25.0)	282 (21.5)	1.33 (1.04–1.72)	0.025	81 (45.3)	0			
140 ≤	54 (11.6)	79 (6.0)	2.20 (1.52–3.18)	3.0×10^−5^	28 (15.6)	0			
DBP, mmHg	79.09±0.56	77.20±0.28	1.02 (1.01–1.03)	0.001^d^	77.24±0.74	67.37±0.29		1.20 (1.16–1.25)	3.4×10^−26^^c^^,^^d^
< 80	329 (70.4)	1,004 (76.5)	1.00		86 (48.0)	500 (100)			
80–90	84 (18.0)	224 (17.0)	1.14 (0.86–1.51)	0.345	64 (35.8)	0		-	
90 ≤	54 (11.6)	85 (6.5)	1.94 (1.35–2.79)	3.5×10^−4^	29 (16.2)	0			
Hb, g/dL^e^	13.80±0.06	13.52±0.04	1.13 (1.05–1.21)	8.4×10^−4^^d^	14.36±0.14	13.47±0.07		1.37 (1.23–1.53)	1.5×10^−8^^d^
13.8–17.2 (12.1–15.1)	353 (75.6)	1,022 (77.8)	1.00		148 (82.7)	410 (82.0)		1.00	
< 13.8 (< 12.1)	110 (23.5)	282 (21.5)	1.13 (0.88–1.45)	0.343	20 (11.2)	73 (14.6)		0.76 (0.45–1.29)	0.307
17.2 < (15.1 <)	4 (0.9)	9 (0.7)	1.29 (0.39–4.20)	0.676	11 (6.1)	17 (3.4)		1.79 (0.82–3.92)	0.143
BUN, mg/dL	14.47±0.17	13.97±0.09	1.04 (1.01–1.07)	0.008^d^	13.92±0.28	13.32±0.16		1.05 (0.99–1.10)	0.060^d^
≤ 20	428 (91.6)	1,235 (94.1)	1.00		168 (93.8)	474 (94.8)		1.00	
20 <	39 (8.4)	78 (5.9)	1.44 (0.97–2.15)	0.072	11 (6.2)	26 (5.2)		1.19 (0.58–2.47)	0.633

Abbreviations: BMI, body mass index, BUN, blood urea nitrogen; CI, confidence interval; DBP, diastolic blood pressure; Hb, hemoglobin; OR, odds ratio; SBP, systolic blood pressure

^a^ Data are shown as the numbers of subjects (percentage) for discrete and categorical variables and mean ± standard error for continuous variables.

^b^ ORs and *P* values were estimated from univariate logistic regression analysis.

^c^ The variables remained significant at *P* value less than 0.05 after backward elimination in a multivariate logistic regression model.

^d^ The ORs, 95% CIs, and *P* values for the continuous variables were estimated using univariate logistic regression analyses.

^e^ Hemoglobin levels in males (females).

### Genome-wide association and replication studies

After elimination of the SNPs in linkage disequilibrium (LD, *r*^*2*^ > 0.8), 374 unique SNPs achieved a significance level of *P* < 0.001 in the KARE GWAS with adjustment for age, sex, and BMI (data not shown). However, none of these SNPs achieved conventional genome-wide significance (*P* < 5×10^−8^). Of the six SNPs with *P* < 1.0×10^−5^, the rs3825435 SNP located on intron of the *FARP1* gene (13q32.2) showed the strongest association with PTB under an additive model (OR = 1.69, 95% CI 1.38–2.07, *P* = 5.3×10^−7^) (Table 2). A total of ten out of the 374 SNPs were associated with PTB at *P* < 0.05, and two SNPs, rs9682385 near the *GADL1* gene (3p23) and rs9787961 near the 5’-UTR of the *CTR9* gene (11p15.3), yielded the strongest associations with PTB in the HEXA dataset (*P* = 0.007 and 0.008, respectively) (Table 3). The joint analysis of data from the KARE and HEXA cohorts did not improve the observed statistical significance but suggested that an intergenic SNP, rs9365798, located 400 kb downstream of the *C6orf118* gene (6q27) was most significantly associated with PTB (OR = 1.59, 95% CI 1.32–1.92, *P* = 2.1×10^−6^). Among the SNPs reported in previous studies, eight SNPs located near or in seven genes were evaluated; however, none of these SNPs showed evidence of association with PTB in either the KARE or HEXA Study datasets (Table B in S1 File). Furthermore, none of the SNPs in the three genes reported to be associated with PTB in recent GWASs, *HLA* class II, *ASAP1*, and *WNT1*, yielded a significant allelic association with PTB in the KARE GWAS (Table C in S1 File). One SNP, rs571110, of the six SNPs with *P* < 1.0×10^−5^ identified in the KARE GWAS (Table 2) showed a statistical power of 86% under a recessive model; however, the current study was underpowered for detection of other SNPs at genome-wide significance level, ranging from 24% (rs9381416) to 68% (rs17394081). Among the ten replicated SNPs that are shown in Table 3, the rs9840514 showed a sufficient statistical power under a recessive model in the joint analysis (99%); other SNPs were underpowered, ranging from 12% (rs9682385) to 72% (rs4348560).

**Table 2:** Results of the genome-wide association study for pulmonary tuberculosis in the KARE Study (*P* < 1×10^−5^).

Gene	Chr.	SNP	Function	Model^a^	N/R	NN/NR/RR^b^		RAF	MLR^c^	
						Cases	Controls	Cases/Controls	OR (95% CI)	*P*
*FARP1*	13q32.2	rs3825435	intron	A	T/C	303/144/20	996/298/18	0.20/0.13	1.69 (1.38–2.07)	5.3×10^−7^
*OXR1*	8q23.1	rs3110431	intron	D	T/C	180/223/54	668/519/126	0.37/0.29	1.70 (1.37–2.12)	2.1×10^−6^
*CLIC5*	6p12.3	rs9381416	intron	A	A/C	76/216/174	320/640/353	0.61/0.51	1.44 (1.23–1.67)	4.0×10^−6^
*KLHL36*	16q24.1	rs2326344	intergenic	A	G/T	203/214/50	726/506/81	0.34/0.25	1.48 (1.25–1.75)	4.9×10^−6^
*IGSF11*	3q13.3	rs571110	intergenic	R	G/A	258/150/47	766/463/59	0.27/0.23	2.56 (1.70–3.85)	6.9×10^−6^
*SRBD1*	2p21	rs17394081	intron	D	C/T	423/43/1	1257/55/1	0.05/0.02	2.61 (1.72–3.98)	7.9×10^−6^

Abbreviations: Chr., chromosome; N/R, non-risk/risk allele; OR, odds ratio; MLR, multiple logistic regression; RAF, risk allele frequency; SNP, single nucleotide polymorphism

^a^ The genetic model that showed the most significant evidence for association with PTB: A, additive; R, recessive.

^b^ NN/NR/RR, the numbers of cases and controls with non-risk homozygote/heterozygote/risk homozygote genotypes, respectively.

^c^ ORs and *P* values were estimated from the MLR model adjusted for age, sex, and BMI.

**Table 3:** Ten replicated SNPs associated with PTB in the KARE and HEXA Studies.

Gene	Chr.	SNP	Function	Model^a^	N/R^b^	KARE		HEXA		Joint Analysis	
						OR (95% CI)^c^	*P*	OR (95% CI)^c^	*P*	OR (95% CI)^c^	*P*
*CDCA7*	2q31.1	rs7594926	intergenic	D	G/A	1.59 (1.22–2.08)	7.6×10^−4^	1.64 (1.05–2.56)	0.030	1.54 (1.23–1.89)	1.4×10^−4^
*GBE1*	3p12.2	rs2307058	intron	R	C/T	1.75 (1.31–2.33)	1.4×10^−4^	1.60 (1.02–2.50)	0.040	1.63 (1.29–2.05)	3.4×10^−5^
*GADL1*	3p23	rs9682385	intergenic	A	G/A	1.34 (1.14–1.58)	5.4×10^−4^	1.45 (1.11–1.91)	0.007	1.31 (1.15–1.50)	7.9×10^−5^
*SPATA16*	3q26.31	rs9840514	intergenic	R	T/C	1.79 (1.27–2.50)	9.5×10^−4^	1.96 (1.16–3.23)	0.011	1.96 (1.16–3.23)	5.4×10^−5^
*C6orf118*	6q27	rs9365798	intergenic	D	T/C	1.61 (1.27–2.04)	7.1×10^−5^	1.49 (1.02–2.17)	0.038	1.59 (1.32–1.92)	2.1×10^−6^
*KIAA1432*	9p24.1	rs4348560	intron	R	G/A	1.79 (1.28–2.50)	6.3×10^−4^	1.75 (1.04–2.94)	0.034	1.69 (1.30–2.22)	1.3×10^−4^
*DMRT2*	9p24.3	rs10738171	intergenic	A	C/T	1.33 (1.12–1.57)	8.9×10^−4^	1.34 (1.03–1.75)	0.029	1.30 (1.14–1.49)	1.3×10^−4^
*CTR9*	11p15.3	rs9787961	Near 5’-UTR	R	C/G	1.67 (1.26–2.21)	3.9×10^−4^	1.83 (1.17–2.85)	0.008	1.64 (1.30–2.06)	2.3×10^−5^
*CCDC67*	11q21	rs3019221	intron	A	G/C	1.61 (1.25–2.07)	2.3×10^−4^	1.50 (1.00–2.23)	0.048	1.56 (1.27–1.91)	2.1×10^−5^
*CDH13*	16q23.3	rs12716963	intron	D	C/T	1.54 (1.19–1.96)	7.7×10^−4^	1.72 (1.12–2.83)	0.012	1.56 (1.27–1.92)	2.6×10^−5^

Abbreviations: Chr., chromosome; CI, confidence interval; HEXA, Health Examinees Study; KARE, Korea Association Resource Study; OR, odds ratio.

^a^ The genetic models that showed the most significant evidence for association with PTB: A, additive; D, dominant; R, recessive.

^b^ N/R, non-risk/risk allele

^c^ ORs and *P* values were estimated from the multiple logistic regression model adjusted for age, sex, and BMI.

### *In silico* functional analysis

Among the ten replicated SNPs, eight SNPs, including six SNPs in strong LD with four genotyped SNPs, located in four genes (*r*^*2*^ > 0.8) fell into different functional categories: transcription factor binding sites (TFBSs), exonic splicing sites (ESSs), miRNA, and nonsynonymous SNPs (nsSNPs) (Table D in S1 File). Specifically, rs2259633 (*CCDC67*, 11q21) have been predicted to be an amino acid substitution (Gln > Lys). This SNP was in strong LD with the SNP rs3019221 identified in the current GWAS (*r*^2^ = 1.00).

### Risk prediction models for pulmonary tuberculosis

**Fig 1:**
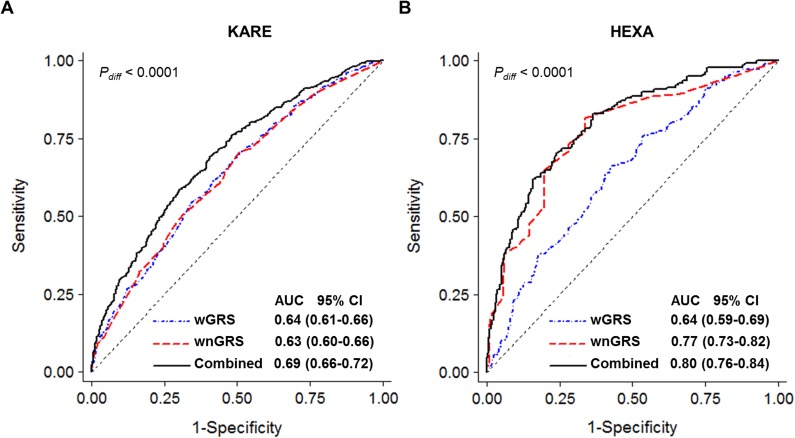
**Comparison of predictability of risk prediction models for tuberculosis in KARE (463 cases and 1,308 controls) (A) and HEXA (142 cases and 490 controls) (B) Studies.** wGRS comprised of ten SNPs replicated in both studies (blue-dotted line), wnGRS comprised of six nongenetic factors, age, sex, body mass index (< 20 kg/m^2^), systolic blood pressure (120 mmHg <), hemoglobin, cigarette smoking (red dashed lines), and the combined model of wGRS and wnGRS (solid black lines).

**Fig 2:**
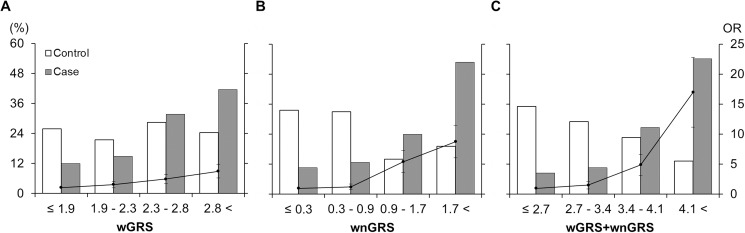
**Comparison of genetic (A), nongenetic (B), genetic plus nongenetic (C) models in 142 cases and 490 controls after removing individuals with missing values from HEXA data.** White and dark-gray bars (left Y-axis) denote the proportions of controls and cases, respectively, in each risk quartile. Black dots and solid lines (right Y-axis) denote odds ratios and their standard errors for each risk quartile group compared to the lowest risk quartile group (X-axis).

The estimated AUC values of the wGRS model composed of the ten SNPs that were validated in the HEXA dataset were approximately 0.64 within both study populations. Data for the missing rate of household income was as high as 17% in the HEXA dataset; thus, this variable was excluded from subsequent analyses. Inclusion of cigarette smoking and alcohol consumption did not improve the predictive ability of the model (AUC, 0.630 to 0.627 in KARE and 0.693 to 0.689 in HEXA); however, the combined model including age, sex, BMI, SBP, Hb, and smoking leads to a much greater AUC increase in the HEXA study (AUC, 0.80; sensitivity 0.83, specificity 0.63, Fig 1B) than in KARE Study (AUC, 0.69; sensitivity 0.70, specificity 0.59, Fig 1A) (Table 4). The wGRS model replicated in HEXA Study captured significantly more risk than did any individual SNP, with 3.7-fold increased risk of PTB identified in the highest risk quartile compared with the lowest risk quartile (*P* = 1.6×10^−5^, Fig 2A). Persons in the highest risk quartile in the combined model were identified to have a higher risk of PTB (OR = 16.98, 95% CI 8.67–33.24, *P* = 1.4×10^−16^, Fig 2C) than the risk that was identified the model in which only conventional risk factors were taken into account (OR = 8.78, 95% CI 4.77–16.14, *P* = 2.8×10^−12^, Fig 2B) (Table E in S1 File).

**Table 4:** Replication of risk models for pulmonary tuberculosis.

Risk model		SNP			AUC (95% CI)^a^		
*P*_*diff*_ < 1×10^−4^^b^		N	wGRS^c^	wnGRS1^d^	wnGRS2^e^	wnGRS3^f^	wnGRS4^g^
*KARE*			0.636 (0.607–0.665)				
Nongenetic model	Cases/Controls, N			467/1313	463/1308	463/1308	463/1305
				0.630 (0.602–0.659)	0.627 (0.598–0.657)	0.634 (0.604–0.663)	0.633 (0.603–0.662)
Combined model	Cases/Controls, N			467/1313	463/1305	463/1308	463/1305
				0.690 (0.663–0.718)	0.684 (0.656–0.712)	0.692 (0.665–0.720)	0.687 (0.659–0.715)
*HEXA*			0.639 (0.587–0.692)				
Nongenetic model	Cases/Controls, N			179/500	177/498	151/498	141/498
				0.693 (0.650–0.736)	0.689 (0.643–0.735)	0.770 (0.726–0.814)	0.780 (0.734–0.825)
Combined model	Cases/Controls, N			170/490	168/490	142/490	132/490
				0.739 (0.697–0.781)	0.736 (0.692–0.780)	0.799 (0.758–0.841)	0.808 (0.765–0.851)

Abbreviations: AUC, area under the receiver operating curve; CI, confidence interval; wGRS weighted genetic risk score; wnGRS, weighted non-genetic risk score.

^a^ AUCs and *P* values were estimated for ROC analyses

^b^ *P*_*diff*_ < 1×10^−4^, AUCs of the three risk score models, wGRS, wnGRS, and wGRS+wnGRS, were significantly different from each other at *P* < 0.0001.

^c^ wGRS, the weighted genetic risk score model composed of the ten replicated SNPs.

^d^ wGRS+wnGRS1, the combined model of wGRS model composed of 10 validated SNPs plus wnGRS1 comprised of age, sex, BMI.

^e^ wnGRS2 was comprised of wnGRS1 plus cigarette smoking, and alcohol consumption.

^f^ wnGRS3 was comprised of wnGRS1 plus cigarette smoking, SBP and Hb.

^g^ wnGRS4 was comprised of wnGRS3 plus alcohol consumption, DBP, and BUN.

## Discussion

Traditional risk factors such as gender (male), aging, low household income, cigarette smoking, alcohol consumption, and high blood pressure, which is one of the risk factors for DM, were consistently found to be associated with PTB risk [15, 21]. Specifically, male, increased age and low BMI were identified as strong predictors of PTB in Korean adult populations. Consistent with previous studies, blood pressure was positively correlated with BMI (*β* = 0.02, 95% CI 0.02–0.03, *P* = 6.4×10^−9^ and *β* = 0.04, 95% CI 0.03–0.05, *P* = 1.5×10^−11^ for SBP and DBP, respectively), while BMI was negatively associated and blood pressure was positively associated with PTB in the current study [2, 21]. Categorization of two continuous predictors, Hb and BUN, made them insignificant. These results support previous findings that categorization of continuous risk factors, especially dichotomization, reduces statistical power and leads to incomplete correction for confounding factors [22]. Thus, baseline BMI, SBP, DBP, HB, and BUN were investigated as continuous variables in multivariate logistic analysis. We cannot, however, rule out potential temporal bias in the estimation of causal effects of the nongenetic factors as prevalent cases were analyzed in the current study.

Recent GWASs have proposed a few candidate genes for PTB; however, most of these SNPs are located in intronic regions, and subsequent studies have not replicated previous findings, except for the *WT1* gene (rs2057178) [5–11]. None of the associations previously identified between PTB and these gene variants were replicated in the evaluated Korean populations; instead, SNPs located in introns of the *SRBD1*, *CLIC5*, *OXR1*, and *FARP1* genes or nearby the genes, *IGSF11* and *KLHL36*, were identified to be associated with PTB at *P* values of less than 1×10^−5^. Three genes, *SRBD1*, *CLIC5*, and *KLHL36*, have been found to be involved in immune responses and reported to be associated with various phenotypes, such as glaucoma, neurological disorders, and carcinoma [23–25]. Dysfunction of the *OXR1* gene resulted in increased oxidative stress associated with neurodegenerative disorders [26]. *IGSF11*-specific activation has been reported to be correlated with HLA-A*0201-restricted cytotoxicy [27]. The role of *KLHL36* gene in human complex disease is unknown. Of ten genes replicated in the HEXA Study dataset, three tumor suppressor genes, *CDCA7*, *KIAA1432*, and *CCDC67*, which were functionally annotated with Polyphen-2 may directly or indirectly play key roles in the development of PTB through immune system and inflammatory responses. The *CDCA7* and *KIAA1432* genes have been reported to be highly overexpressed in lung cancer cells [28, 29]. *CCDC67* mRNA, which encodes a 604 amino acid protein containing a coiled-coil domain, was associated with down-regulation in various tumor tissues such as lung, cervix, and gastric cancers [30]. The *CDH13* polymorphisms play critical roles in metabolic processes and in the development of metabolic-related diseases and lung diseases such as chronic obstructive pulmonary disease [31, 32]. In most previous PTB GWASs, nongenetic covariates were not considered; however, we could not replicate the associations reported in these studies, even in our unadjusted model. This lack of replication may be due to considerable genetic, phenotypical and environmental diversity among study populations; more fundamentally, however, PTB may occur as a result of a complex host-pathogen-environment interplay and polygenic pathways including many genetic variants, each with a relatively small effect [33].

Previous studies have recognized that genetic risk models for complex diseases developed based on specific populations are unlikely to be applicable to other populations because the small effect sizes of common alleles tend to vary based on population composition and may even be identified as having opposing effects in different populations, and risk estimates for some variants could be imprecise, as various characteristics of study subjects potentially confound genetic associations [34]. This study provided important insights into the identification of susceptibility genes for PTB. Overall, we improved the discriminatory ability of PTB risk models by including both genetic and conventional risk factors. The risk model that comprised ten replicated SNPs was found to have similar AUCs in both study datasets (0.636 and 0.639, respectively). Inclusion of six conventional risk factors (i.e., age, sex, BMI, SBP, Hb, and cigarette smoking) resulted in a greater improvement in PTB risk prediction in the HEXA Study than in the KARE Study (AUC, 0.80 vs. 0.69), which was likely due to the stronger effects of the nongenetic factors observed in the HEXA dataset (e.g., OR for men, 4.2 vs. 2). However, addition of three risk factors, alcohol consumption, DBP, and BUN did not significantly improve the AUC of the model, wGRS+wnGRS3 (Table 4). The best prediction of individual disease risk is achieved using a large number of predictors; however, given several models with similar predictive accuracy, the simplest model with fewer predictors is considered to be more efficient and cost-effective by collecting relevant minimum information on patients [35]. Although their signature was not found to be sufficiently predictive, a recent African study prospectively identified RNA markers to predict progression to active PTB among latent PTB patients (Sensitivity 53.7%, Specificity 82.8%) [36]. Future studies are warranted to perform validations of the risk models in independent populations with substantially larger samples, and further prospective investigations are needed. Clearly, future risk models with clinical utility in preventing active PTB will need to include susceptibility variants with potent discriminatory ability based on whole genome sequencing and omics data integration; however, these models will likely have limited applicability in the near future due to cost constraints and technical limitations [34, 37]. Gene-gene interactions or epistasis are a potential source of missing heritability and new strategies for modeling epistasis to improve the predictability of PTB risk may warrant further examination in future studies.

The current study consisted of 646 PTB patients and 1,813 health Koreans, aged 40 years and above, selected from two population based cohorts. The present study may have been underpowered to detect genetic variants for PTB susceptibility with weak or modest effect sizes at a genome-wide level of significance; hence further studies with large PTB cases are warranted to validate our findings. Given the importance of age at PTB onset, future studies in patients aged younger than 40 years will be important to decipher the genetic basis for PTB arising at young age. HIV infection is the most potent risk factor for developing active PTB [3]. In the current study, however, none of the participant reported being HIV infected and the prevalence of HIV was very low in South Korea, about 4.2 per 100,000 people in 2009 [38]. The genetic risk prediction models developed in this study seemed to have limited predictive power for clinical use; however, the performance of risk prediction based on the results of numerous models suggested important insights regarding ongoing attempts to identify individuals susceptible to PTB in the general population. Firstly, models considering polygenic inheritance and evaluating the role of and interactions between replicated variants influencing the metabolic, inflammatory, and immune pathways in the pathogenesis of infectious disease may better explain an individual’s risk of developing PTB. Secondly, we found that gender and BMI were the strongest predictors of PTB and validated the effects of a number of nongenetic risk factors in an independent Korean population. Finally, our strategy of constructing risk prediction models for PTB by combining genetic and nongenetic risk factors may ultimately result in better risk prediction. These findings pave the way for the development of models to identify susceptible individuals and prevent their progression to active PTB.

## Supporting information

S1 File Supplementary Tables. Table A, Multivariate logistic regression analysis and associations between baseline characteristics and pulmonary tuberculosis in the KARE and HEXA Studies. Table B, Comparison of association results of previous reported SNPs with the current study. Table C, Association results of the SNPs near or in three reported genes, *HLA* class II, *ASAP1*, and *WNT1*, in the KARE GWAS. Table D, Functional annotation of eight SNPs, including six SNPs in strong linkage disequilibrium (*r*^2^ > 0.8 and average *r*^2^ = 0.95) with four genotyped SNPs identified in the KARE Study (*P* < 0.001). Table E, Associations between risk score quartiles and tuberculosis in KARE and HEXA Studies.
(DOCX)
