# Supplementary material for: Risk prediction of pulmonary tuberculosis using genetic and conventional risk factors in adult Korean population
Source: PLoS One. 2017 Mar 29;12(3):e0174642. doi: 10.1371/journal.pone.0174642 (PMC5371343; doi:10.1371/journal.pone.0174642)
Supplement: S1 File — Table A, Multivariate logistic regression analysis and associations between baseline characteristics and pulmonary tuberculosis in the KARE and HEXA Studies. Table B, Comparison of association results of previous reported SNPs with the current study. Table C, Association results of the SNPs near or in three reported genes, HLA class II, ASAP1, and WNT1, in the KARE GWAS. Table D, Functional annotation of eight SNPs, including six SNPs in strong linkage disequilibrium (r2 > 0.8 and average r2 = 0.95) with four genotyped SNPs identified in the KARE Study (P < 0.001). Table E, Associations between risk score quartiles and tuberculosis in KARE and HEXA Studies. (DOCX) [file pone.0174642.s001.docx]

**Supplementary Table A** Multivariate logistic regression analysis and associations between baseline characteristics and pulmonary tuberculosis in the KARE and HEXA Studies

|  | KARE^b^ | | | HEXA^b^ | | |
| --- | --- | --- | --- | --- | --- | --- |
| Characteristics^a^ | OR | 95% CI | *P* | OR | 95% CI | *P* |
| Male, N (%) | 2.13 | 1.41-3.21 | 3.0×10^-9c^ | 2.69 | 1.23-5.89 | 0.013^c^ |
| Age, N (%) |  |  |  |  |  |  |
| < 50 | 1.00 | - | - | 1.00 | - | - |
| 50 ≤ | 1.46 | 1.13-1.88 | 0.004^c^ | 1.50 | 0.87-2.58 | 0.143 |
| Household income (million won), N (%) |  |  |  |  |  |  |
| < 100 | 1.00 | - | - | 1.00 | - | - |
| 100 ≤ | 0.82 | 0.62-.107 | 0.167 | 0.67 | 0.31-1.45 | 0.311 |
| Cigarette smoking, N (%) |  |  |  |  |  |  |
| Nonsmoker | 1.00 | - | - | 1.00 | - | - |
| Ex-smoker | 0.97 | 0.65-1.43 | 0.872 | 0.98 | 0.42-2.27 | 0.957 |
| Current smoker | 0.75 | 0.52-1.08 | 0.126 | 0.86 | 0.35-2.10 | 0.742 |
| Alcohol consumption, N (%) |  |  |  |  |  |  |
| Nondrinker | 1.00 | - | - | 1.00 | - | - |
| Ex-drinker | 1.26 | 0.78-2.04 | 0.340 | 1.12 | 0.33-3.78 | 0.849 |
| Current drinker | 1.14 | 0.87-1.50 | 0.326 | 0.89 | 0.51-1.57 | 0.694 |
| BMI, kg/m^2^ | 0.85 | 0.81-0.88 | 3.0×10^-14c^ | 0.84 | 0.76-0.94 | 0.001^c^ |
| SBP, mmHg | 1.01 | 0.99-1.02 | 0.075 | 1.15 | 1.10-1.20 | 7.3×10^-10c^ |
| DBP, mmHg | 1.00 | 0.98-1.02 | 0.903 | 1.07 | 1.01-1.13 | 0.008^c^ |
| Hb, g/dL | 0.93 | 0.87-1.07 | 0.487 | 0.99 | 0.82-1.19 | 0.908 |
| BUN, mg/dL | 1.02 | 0.98-1.05 | 0.302 | 0.98 | 0.91-1.06 | 0.622 |

Abbreviations: BMI, body mass index, BUN, blood urea nitrogen; CI, confidence interval; DBP, diastolic blood pressure; Hb, hemoglobin; OR, odds ratio; SBP, systolic blood pressure.

^a^ Data are shown as the numbers of subjects (percentage) for discrete and categorical variables and mean ± standard error for continuous variables.

^b^ ORs, 95% CIs, and *P* values were estimated from multiple logistic regression analysis.

^c^ The variables remained significant at *P* value less than 0.05 after backward elimination in a multivariate logistic regression model.

**Supplementary Table B** Comparison of association results of previous reported SNPs with the current study

| Gene | Chr. | Reported | Function | NE/E | KARE^a^ | |  | HEXA^a^ | | Previous study | | | | |
| --- | --- | --- | --- | --- | --- | --- | --- | --- | --- | --- | --- | --- | --- | --- |
|  |  | SNP |  |  | OR | *P* |  | OR | *P* | Ethnicity | Case/Control, N | OR | *P* | Reference |
| *Genome-wide association study* | | |  |  |  |  |  |  |  |  |  |  |  |  |
| *ASAP1* | 8q24.1 | rs1469288^b^ | intron | T/G | 0.98 | 0.876 |  | 0.95 | 0.198 | RUS | 6,169/7,736 | 0.84 | 1.1×10^-10^ | Curtis et al. 2015 |
|  | -q24.2 | rs1017281^b^ | intron |  |  |  |  |  |  | RUS | 6,436/8,064 | 0.85 | 3.7×10^-10^ | Curtis et al. 2015 |
|  |  | rs2033059^b^ | intron |  |  |  |  |  |  | RUS | 6,454/7,958 | 0.84 | 4.3×10^-11^ | Curtis et al. 2015 |
|  |  | rs4733781^b^ | intron |  |  |  |  |  |  | RUS | 6,432/8,082 | 0.84 | 2.6×10^-11^ | Curtis et al. 2015 |
| *WT1* | 11p13 | rs2057178^b^ | intron | G/A | - | - |  | 0.89 | 0.752 | S.AFR | 642/91 | 0.62 | 2.7×10^−6^ | Chimusa et al. (2014) |
|  |  |  |  |  |  |  |  |  |  | GHA | 2127/5636 | 0.77 | 2.6×10^-9^ | Thye et al. (2012) |
|  |  |  |  |  |  |  |  |  |  | GMB | 1207/1349 | 0.8 | 4.9×10^-4^ | Thye et al. (2012) |
|  |  |  |  |  |  |  |  |  |  | IND | 1025/983 | 0.84 | 0.099 | Thye et al. (2012) |
|  |  |  |  |  |  |  |  |  |  | RUS | 4441/5874 | 0.91 | 0.020 | Thye et al. (2012) |
|  |  |  |  |  | - | - |  |  |  | Multi-ethnic | 8821/13859 | NR | 2.6×10^-11^ | Thye et al. (2012) |
| *LOC105372021* | 18q11.2 | rs4331426 | intron | A/G | 1.02 | 0.951 |  | 1.57 | 0.197 | AFR | 3632/7501 | 1.19 | 6.8×10^-9^ | Thye et al. (2010) |
| *Candidate gene study* | |  |  |  |  |  |  |  |  |  |  |  |  |  |
| *WT1* | 11p13 | rs2057178^b^ | intron | G/A | - | - |  | 0.89 | 0.752 | CHB | 763/763 | 0.4 | 6.0×10^-4^ | Chen et al. (2016) |
| *TLR1* | 4p14 | rs4833095^b^ | N248S | T/C | - | - |  | 1.04 | 0.739 | AFR.AMR | 339/194 | 1.63 | 0.009 | Ma et al. (2007) |
| *NOS2A* | 17q11.2 | rs7215373^b^ |  | G/T | 1.01 | 0.868 |  | 1.07 | 0.625 | AFR.AMR | 279/166 | 1.67 | 0.004 | Velez et al. (2009) |
|  |  | rs8078340^b^ |  | G/A | 0.96 | 0.903 |  | 0.53 | 0.318 | S.AFR | 431/482 | 1.4 | 0.011 | Moller et al. (2009) |
| *TLR8* | Xp22 | rs3764880^b^ | Met1Val | C/T | 0.97 | 0.836 |  | 1.37 | 0.090 | IND | 375/387 | 1.8 | 0.007 | Davila et al. (2008) |
|  |  |  |  |  |  |  |  |  |  | RUS | 1837/1779 | 1.2 | 0.030 | Davila et al. (2008) |

Abbreviations: AFR, African; AFR.AMR, African American; CHB, Chinese; Chr, chromosome; NE/E, non-effect/effect allele; GHA, Ghanaian; GMB, Gambian; IND, Indonesian; OR, odds ratio; RUS, Russian; S.AFR, South African; SNP, single nucleotide polymorphism.

^a^ ORs and *P* values were estimated from the multiple logistic regression analyses adjusted for age, sex, residence, and BMI in KARE and adjusted for age, sex, and BMI in HEXA.

^b^ SNPs being in linkage disequilibrium (LD) based on Japanese (JPT) and/or Chinese (CHB) HapMap database (*r*^2^ > 0.8) were analyzed in the current study: rs1469288, rs1017281, rs2033059, and rs4733781 with rs11774947 (*r*^2^ > 0.82 in both JPT and CHB); rs2057178 with rs17673047 (*r*^2^ = 1.0 in CHB); rs4833095 with rs2101521 (*r*^2^ = 0.81 in JPT and CHB); rs7215373 with rs9303680 (*r*^2^ > 0.90 in JPT and CHB); rs8078340 with rs16966434 (*r*^2^ = 1.0 in CHB); rs3764880 with rs3788935 (*r*^2^ = 1.0 in JPT and CHB).

**Supplementary Table C** Association results of the SNPs near or in three reported genes, *HLA* class II, *ASAP1*, and *WNT1*, in the KARE GWAS.

| Gene | SNP | Function | M/m | OR^a^ | *P*^a^ | Gene | SNP | Function | M/m | OR^a^ | *P*^a^ |  |
| --- | --- | --- | --- | --- | --- | --- | --- | --- | --- | --- | --- | --- |
| ***HLA class II*** | 6p21.32 |  |  |  |  | ***ASAP1*** | 8q24.1-q24.2 |  |  |  |  |  |
| *HLA-DRA* | rs9405035 | Near UTR-5 | C/T | 0.84 | 0.202 |  | rs74786617^b^ | Near UTR-3 | A/G | 1.21 | 0.319 |  |
|  | rs3129872 | Near UTR-5 | T/A | 0.97 | 0.749 |  | rs4236749 | UTR-3 | C/T | 0.98 | 0.784 |  |
|  | rs9268645 | Intron | C/G | 0.96 | 0.639 |  | rs2045093^b^ | Intron | T/C | 0.97 | 0.772 |  |
|  | chr6:32409242 | Intron | C/A | 1.12 | 0.192 |  | rs749126 | Intron | C/A | 0.97 | 0.770 |  |
|  | rs3177928 | UTR-3 | C/T | 0.92 | 0.586 |  | rs7003309 | Intron | A/C | 0.93 | 0.467 |  |
|  | rs7194 | UTR-3 | T/C | 1.11 | 0.229 |  | rs6984815 | Intron | G/T | 1.14 | 0.410 |  |
|  | rs1051336 | UTR-3 | C/T | 1.18 | 0.097 |  | rs7838372^b^ | Intron | G/A | 1.03 | 0.892 |  |
| *HLA-DQA1* | rs9272219 | Intron | G/T | 1.18 | 0.111 |  | rs16904220^b^ | Intron | G/T | 1.18 | 0.425 |  |
|  | rs9272346 | Intron | A/G | 0.98 | 0.769 |  | rs9297805 | Intron | C/T | 0.95 | 0.562 |  |
| *HLA-DQA2* | rs9276429^b^ | Intron | A/G | 1.08 | 0.427 |  | rs2554374 | Intron | T/C | 1.03 | 0.763 |  |
|  | rs2239800 | Intron | A/G | 1.02 | 0.803 |  | rs1978551 | Intron | G/G | 1.17 | 0.310 |  |
|  | rs9276435 | Intron | C/T | 1.17 | 0.478 |  | rs7839523 | Intron | T/G | 0.92 | 0.352 |  |
| *HLA-DQB2* | rs2051549^b^ | Intron | A/G | 1.09 | 0.369 |  | rs11774947^b^ | Intron | A/G | 0.96 | 0.612 |  |
|  | rs6903130^b^ | Near UTR-5 | A/G | 1.02 | 0.788 |  | rs16904239^b^ | Intron | G/T | 0.97 | 0.734 |  |
| *HLA-DMB* | chr6:32901934 | Near UTR-3 | G/A | 0.87 | 0.304 |  | rs1426102b | Intron | G/C | 0.97 | 0.783 |  |
|  | rs151719 | Intron | A/G | 1.04 | 0.681 |  | rs2943086 | Intron | G/T | 0.89 | 0.229 |  |
| *HLA-DMA* | chr6:32912588 | Intron | C/A | 1.41 | 0.422 |  | rs2670882 | Intron | T/C | 1.02 | 0.921 |  |
|  | chr6:32917857 | Intron | C/T | 0.98 | 0.835 |  | rs2791349^b^ | Intron | A/G | 1.11 | 0.508 |  |
|  | chr6:32922459 | Intron | C/T | 0.99 | 0.938 |  | rs3924416 | Intron | T/G | 1.30 | 0.061 |  |
|  | chr6:32938199 | Intron | G/A | 1.55 | 0.167 |  | rs4733575 | Intron | C/T | 0.93 | 0.454 |  |
| *HLA-DOA* | chr6:32972207 | UTR-3 | G/A | 1.62 | 0.166 |  | rs3924865^b^ | Intron | T/A | 1.14 | 0.123 |  |
|  | chr6:32972642^b^ | UTR-3 | G/A | 1.08 | 0.411 |  | rs6470818 | Intron | G/T | 1.10 | 0.486 |  |
|  | chr6:32973743^b^ | UTR-3 | A/G | 0.97 | 0.842 |  | rs11775398 | Intron | A/G | 1.19 | 0.138 |  |
|  | rs3129302 | UTR-3 | C/T | 0.79 | 0.467 |  | rs115592734^b^ | Near UTR-5 | A/G | 1.31 | 0.144 |  |
|  | rs399604^b^ | Intron | A/G | 0.99 | 0.950 | ***WT1*** | 11p13 |  |  |  |  |  |
| *HLA-DPA1* | rs3077 | UTR-3 | C/T | 0.95 | 0.498 |  | rs5030316^b^ | UTR-3 | T/G | 0.85 | 0.568 |  |
|  | chr6:33042551 | Intron | G/A | 1.03 | 0.717 |  | rs5030311 | Intron | G/A | 0.68 | 0.165 |  |
| *HLA-DPB1* | chr6:33045500 | Intron | A/G | 1.05 | 0.562 |  | rs5030310^b^ | Intron | T/A | 0.94 | 0.442 |  |
|  | rs1431403^b^ | Intron | C/T | 0.92 | 0.330 |  | rs2900740 | Intron | G/T | 1.06 | 0.784 |  |
|  | chr6:33055899^b^ | Near UTR-3 | G/A | 0.99 | 0.969 |  | rs7110547^b^ | Intron | G/C | 0.94 | 0.525 |  |
| *HLA-DPB2* | chr6:33079766^b^ | Near UTR-5 | A/G | 1.02 | 0.831 |  | rs7936152^b^ | Intron | G/A | 1.10 | 0.584 |  |
|  |  |  |  |  |  |  | rs5030141 | Intron | T/G | 0.97 | 0.701 |  |
|  |  |  |  |  |  |  | rs3809060^b^ | Near UTR-5 | T/C | 0.94 | 0.524 |  |

Abbreviations: Chr, chromosome; M/m, major/minor allele; OR, odds ratio; UTR, untranslated region.

^a^ ORs and p-values were estimated by multiple logistic regression analyses after adjusting for age, sex, residence, and body mass index.

^b^ SNPs in linkage disequilibrium (*r^2^*>0.8) with each SNP were excluded from subsequent analyses.

**Supplementary Table D** Functional annotation of eight SNPs, including six SNPs in strong linkage disequilibrium (*r^2^* > 0.8 and average *r*^2^ = 0.95) with four genotyped SNPs identified in the KARE Study (*P* < 0.001)

| Gene | Chr | KARE | LD-SNPs^a^ | TFBS | ESE or ESS | miRNA | nsSNP | Polyphen-2 |
| --- | --- | --- | --- | --- | --- | --- | --- | --- |
| *CDCA7* | 2q31.1 | rs7594926 |  | Y | -- | -- | -- | -- |
|  |  |  | rs6433416 | Y | -- | -- | -- | -- |
| *KIAA1432* | 9p24 | rs4348560 |  | -- | -- | -- | -- | -- |
|  |  |  | rs10975309 | Y | -- | -- | -- | -- |
| *CTR9* | 11p15.3 | rs9787961 |  | Y | -- | -- | -- | -- |
|  |  |  | rs2279696 | Y | -- | -- | -- | -- |
| *CCDC67* | 11q21 | rs3019221 |  | -- | -- | -- | -- | -- |
|  |  |  | rs2259633 | -- | Y | -- | Q504K | -- |
|  |  |  | rs2446061 | -- | -- | Y | -- | -- |
|  |  |  | rs2658777 | -- | -- | Y | -- | -- |

Abbreviations: Chr, chromosome; ESE, exonic splicing enhancer; ESS, exonic splicing silencer; miRNA, micro ribonucleic acid; nsSNP, non-synonymous single nucleotide polymorphism; TFBS, transcription factor binding site.

^a^ LD-SNP, SNPs in LD with one of the four SNPs identified in the KARE Study.~~.~~

**Supplementary Table E** Associations between risk score quartiles and tuberculosis in KARE and HEXA Studies

|  |  | KARE |  |  |  | HEXA |  |
| --- | --- | --- | --- | --- | --- | --- | --- |
| Risk model^a^ | Case/Control, N (%) | OR (95% CI) | *P* |  | Case/Control, N (%) | OR (95% CI) | *P* |
| wGRS^b^ |  |  |  |  |  |  |  |
| ≤ 1.9 | 55 (12)/324 (25) | Reference |  |  | 17 (12)/127 (26) | Reference |  |
| 1.9 -­ 2.3 | 75 (16)/290 (22) | 1.52 (1.04-2.23) | 0.031 |  | 21 (15)/105 (22) | 1.49 (0.75-2.98) | 0.254 |
| 2.3 ­- 2.8 | 159 (34)/397 (30) | 2.36 (1.68-3.31) | 7.4×10^-7^ |  | 45 (32)/139 (28) | 2.42 (1.32-4.44) | 0.004 |
| 2.8 < | 174 (38)/297 (23) | 3.45 (2.45-4.86) | 1.2×10^-12^ |  | 59 (41)/119 (24) | 3.70 (2.04-6.71) | 1.6×10^-5^ |
| wnGRS^c^ |  |  |  |  |  |  |  |
| ≤ 0.1 | 46 (10)/293 (22) | Reference |  |  | 15 (10)/165 (34) | Reference |  |
| 0.1 ­- 0.7 | 85 (18)/315 (24) | 1.72 (1.16-2.54) | 0.007 |  | 18 (13)/162 (33) | 1.22 (0.60-2.51) | 0.584 |
| 0.7 ­- 1.3 | 112 (24)/323 (25) | 2.21 (1.51-3.22) | 4.0×10^-5^ |  | 34 (24)/69 (14) | 5.42 (2.78-10.59) | 7.5×10^-7^ |
| 1.3 < | 220 (48)/377 (29) | 3.72 (2.61-5.29) | 2.9E-13 |  | 75 (53)/94 (19) | 8.78 (4.77-16.14) | 2.8×10^-12^ |
| wGRS+wnGRS |  |  |  |  |  |  |  |
| ≤ 2.6 | 46 (10)/370 (28) | Reference |  |  | 12 (8)/172 (35) | Reference |  |
| 2.6 ­- 3.2 | 78 (17)/331 (25) | 1.90 (1.28-2.81) | 0.001 |  | 15 (11)/142 (29) | 1.51 (0.69-3.34) | 0.304 |
| 3.2 ­- 3.9 | 136 (29)/347 (27) | 3.15 (2.19-4.54) | 7.0×10^-10^ |  | 38 (27)/111 (23) | 4.91 (2.46-9.80) | 6.5×10^-6^ |
| 3.9 < | 203 (44)/260 (20) | 6.28 (4.39-8.98) | 6.6×10^-24^ |  | 77 (54)/65 (13) | 16.98 (8.67-33.24) | 1.4×10^-16^ |

Abbreviations: CI, confidence interval; OR, odds ratio; wGRS, weighted genetic risk score; wnGRS, weighted non-genetic risk score.

^a^Risk score models of wGRS, wnGRS, and wGRS+wnGRS were stratified into four quartiles (*i.e*., 0-25, 25-50, 50-75, and 75-100%).

^b^wGRS is comprised of ten SNPs replicated in both KARE and HEXA Studies.

^c^wnGRS is comprised of six non-genetic factors, i.e., age, sex, BMI, SBP, Hb, cigarette smoking.
